# Efficient Crystallization of Apo Sirt2 for Small-Molecule Soaking and Structural Analysis of Ligand Interactions

**DOI:** 10.1021/acs.jmedchem.4c02896

**Published:** 2025-05-20

**Authors:** Florian Friedrich, Matthias Schiedel, Sören Swyter, Lin Zhang, Wolfgang Sippl, Mike Schutkowski, Oliver Einsle, Manfred Jung

**Affiliations:** † Institute of Pharmaceutical Sciences, 9174 University of Freiburg, Freiburg 79104, Germany; ‡ Institute of Medicinal and Pharmaceutical Chemistry, 26527 Technische Universität Braunschweig, Braunschweig 38106, Germany; § Institute of Biochemistry, 9174 University of Freiburg, Freiburg 79104, Germany; ∥ Faculty of Synthetic Biology, Shenzhen University of Advanced Technology, Shenzhen 518107, China; ⊥ Department of Medicinal Chemistry, 9176 Institute of Pharmacy, Martin-Luther-University of Halle-Wittenberg, Halle 06120, Germany; # Department of Enzymology, Charles Tanford Protein Center, Institute of Biochemistry and Biotechnology, Martin-Luther-University Halle-Wittenberg, Halle 06120, Germany

## Abstract

The selectivity pocket is a key binding site for inhibitors of the NAD^+^-dependent lysine deacylase Sirtuin 2 (Sirt2), a promising drug target in diseases like cancer. While small-molecule soaking can advance inhibitor development, the selectivity pocket is absent in available Sirt2 apo structures, and existing soaking systems like Sirt2–ADPribose (ADPR) suffer from unfavorable crystal packing that hinders ligand binding. We developed a method to rapidly generate high-quality Sirt2 apo crystals with an open selectivity pocket, suitable for high-throughput soaking. The induced-fit pocket forms upon seeding with a Sirtuin Rearranging ligand (SirReal) and is retained in the ligand-free apo structure. Screening the Maybridge Ro3-fragment library using a fluorescence polarization assay yielded three novel Sirt2-fragment-inhibitor structures. Additionally, our Sirt2 apo crystals can accommodate ligands at the acyl-lysine channel entrance and the cofactor binding site, as confirmed by binding of the peptide inhibitor KT9 and NAD^+^, facilitating SAR studies and inhibitor optimization.

## Graphical abstract

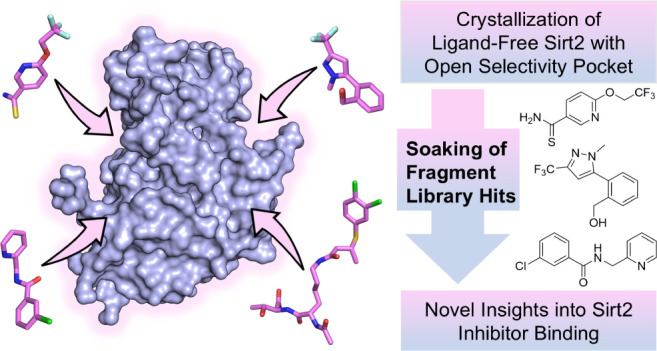

## Introduction

Sirtuins are a unique class of NAD^+^-dependent lysine deacylases, with seven known isotypes (Sirt1–7) in humans. These isotypes share a highly conserved catalytic domain but differ in cellular localization and substrate specificity.^1^

Sirtuin 2 (Sirt2) is primarily cytoplasmic but can shuttle into the nucleus.^2^ It has a broad substrate scope, deacetylating histones (e.g., H4K16 and H3K18)^3,4^ and nonhistone proteins such as α-tubulin,^5^ FOXO3,^6^ GAPDH,^7^ and p300.^8^ Sirt2 also cleaves off long-chain fatty acyl groups (e.g., myristoyl groups),^9,10^ with identified substrates including KRas4a,^11^ RalB,^12^ and ARF6.^13^ Given its wide range of substrates, Sirt2 regulates key cellular pathways, such as mitosis, metabolism, aging, inflammation, and gene transcription.^7,14–17^ Dysregulation of Sirt2 is implicated in numerous diseases, including neurological and metabolic disorders, as well as cancer, which makes Sirt2 a potential drug target.^15,18–20^ Consequently, understanding the unique structural properties of Sirt2 is crucial for designing potent and selective inhibitors.

Sirt2 consists of a Rossmann-fold domain, which accommodates the NAD^+^ binding site, and a smaller, flexible Zn^2+^-binding domain (Figure 1A).^21^ These domains are separated by a hydrophobic channel that facilitates acyl-lysine substrate binding. The deacylation mechanism occurs within this highly conserved catalytic core, which is induced through the closure of the Zn^2+^-binding domain, resulting in a compression of the acyl-lysine channel. The binding of long-chain acyl-lysine substrates, which show a much higher affinity toward Sirt2 than acetylated substrates, induces the formation of a hydrophobic, Sirt2 specific subpocket at the end of the acyl-lysine channel.^9,10^ In 2015, our group identified this pocket and termed it “selectivity pocket,” in the course of the discovery of the Sirt2-selective inhibitor **SirReal2** (Figure 1B).^22^ **SirReal2** opens this pocket primarily through the reorientation of the hinge region consisting of amino acids 138–143 and further locks Sirt2 in a catalytically inactive opened state. The inhibitor class was further improved toward more potent scaffolds, especially with the implementation of a triazole residue that forms specific interactions with Arg97 that led to increased affinities (e.g., **Mz242**, Figure 1B). This in turn allowed the development of cellular probes such as biotinylated or fluorescent inhibitors and the first proteolysis targeting chimeras (PROTACs) for any amidohydrolase, including histone deacetylases.^23–26^

**Figure 1:**
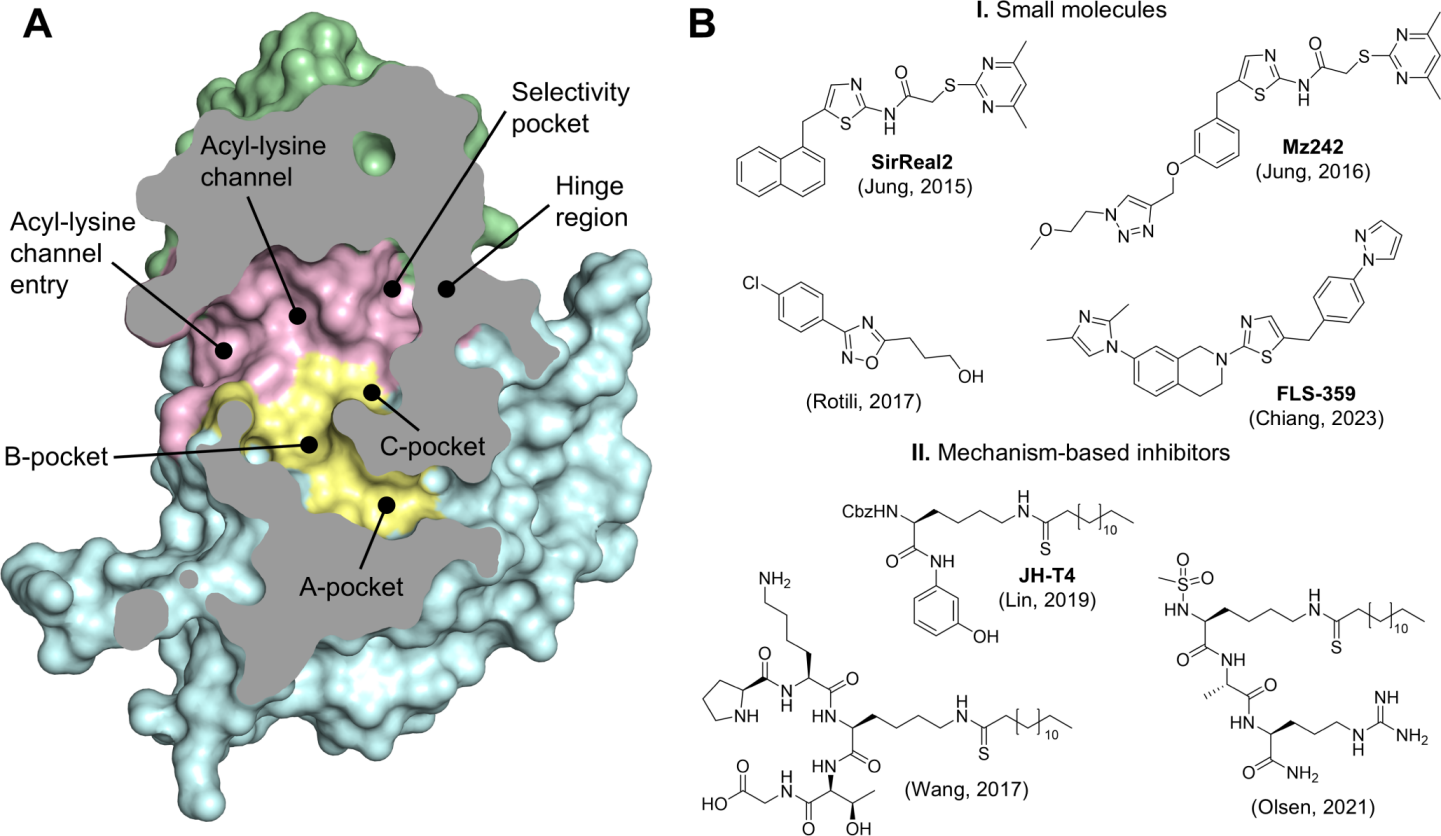
Overall structure of Sirt2 and chemical structures of selected Sirt2 inhibitors. (A) Structural features of *h*Sirt2 (structure from PDB 4RMG) shown as cross-section surface representation. Highlighted are the Rossmann-fold domain (pale cyan) with its NAD^+^ binding site (A-, B- and C-pocket, pale yellow), the Zn^2+^-binding domain (pale green) and the acyl-lysine channel (light pink) located at the interface of the two domains. (B) A selection of published Sirt2 inhibitors. (I) Small molecule Sirt2 inhibitors SirReal2 ^22^ and the improved analogue **Mz242**,^23^ a 1,2,4-oxadiazole inhibitor^38^ and **FLS-359**.^31^ (II) Mechanism-based inhibitors containing a thioamide warhead.^30,34,35^

Since then, structure-based drug design has yielded numerous Sirt2 inhibitors, offering valuable insights into their binding mechanisms. Many of these inhibitors exploit the opening of the selectivity pocket.^27–33^ The structural portfolio of Sirt2 inhibitors is diverse, ranging from small molecules and mechanism-based peptide-like inhibitors,^30,34,35^ (Figure 1B) to chimeric scaffolds^32^ and cyclic peptides.^36,37^ While most Sirt2-inhibitor complexes have been obtained through cocrystallization experiments, only few costructures with soaked molecules exist.

Successful examples include Sirt2-ADPR crystals that were used to solve structures with indole-based (**EX243**, PDB 5D7P, see Figure S1A; **CHIC35**, PDB 5D7Q)^39^ and 1,2,4-oxadiazole-based inhibitors^38^ (Figures 1B and S1A). However, due to disadvantageous crystal contacts, the acyl-lysine channel entry is partially blocked by a neighboring Sirt2 molecule, in which Leu297 acts as a pseudosubstrate and induces the closure of the Zn^2+^–binding domain (Figure S2B,C).^39,40^ This prohibits to use this system as a broad Sirt2 soaking platform. In other studies NAD^+^ was soaked into Sirt2–p53KAc (PDB 2H4F)^41^ and Sirt2–TM (Figure S1B, PDB 4X3O)^34^ crystals, leading to the structural evaluation of different catalytic intermediates.

Herein, we developed an original soaking system using ligand-free *h*Sirt2 apo crystals with an open selectivity pocket and favorable crystal contacts. Our new approach allows the efficient characterization of a wide range of Sirt2 inhibitor and substrate binding modes, and facilitates structural analysis of weak-binding molecules (e.g., initial screening hits), which are challenging to study using conventional cocrystallization methods.

## Results

### Generation of Apo *h*Sirt2 Crystals for Soaking Experiments and Proof of Concept

For the generation of ligand-free *h*Sirt2 apo crystals featuring an open selectivity pocket, crystals of the Sirt2–**SirReal2**–NAD^+^ and Sirt2–**Mz242** complex were generated (see Materials and Methods section for crystallization details).^42^ Crystallization trials with apo Sirt2, combined with microseed-matrix screening (MMS)^43^ using either Sirt2–**SirReal2**–NAD^+^ or Sirt2–**Mz242** crystals, yielded highly reproducible and well-diffracting Sirt2 apo crystals, in which Sirt2 adapts to the *P*2_1_ space group and the conformation of the structures in the ligand-bound state (PDB 9FDR, final resolution of 1.25 Å). In this space group, the packing of Sirt2 results in crystal contacts that neither occlude the entrance of the acyl-lysine channel nor the NAD^+^ binding site. Comparison of the ligand-free Sirt2 apo structure (PDB 9FDR) with Sirt2–**Mz242** (PDB 8OWZ) revealed an almost identical orientation of the main chain C_α_-atoms (rmsd of 0.308 Å, see Figure S3A), but notable differences in the orientation of Phe96 and Arg97 within the acyl-lysine channel, which form strong interactions with **Mz242**. In the novel apo structure, the selectivity pocket is opened due to the conformational adaptation of Sirt2 to **Mz242** binding, distinguishing it from the previously deposited Sirt2 apo structure (PDB 3ZGO).^42^ A PEG molecule binding inside the acyl-lysine channel stabilizes this conformation. It is absent in PDB 3ZGO (Figure 2A), but has been observed in other sirtuin structures, such as Sirt3 (PDB 5D7N).^39^ The flexible cofactor binding loop comprising of amino acids 90–110 shows well resolved electron density. All aforementioned features make these crystals ideal for soaking experiments with small molecules occupying the acyl-lysine channel, selectivity pocket or NAD^+^ binding site of Sirt2. As a proof-of-concept, we first decided to soak the commercially available Sirt2 inhibitor **SirReal2** into the Sirt2 crystals to obtain the Sirt2–**SirReal2** structure (Figure 2B, PDB 9FDS). So far, **SirReal2** has only been cocrystallized as a ternary complex of Sirt2 together with either an H3-AcLys peptide or NAD^+^, but never as a binary complex with Sirt2 alone.^22^ Superimposition with the Sirt2–**SirReal2**–NAD^+^ structure (PDB 4RMG) resulted in a very well alignment of the protein backbone (rmsd of 0.401 Å). In the two structures, the binding of **SirReal2** shows high similarity, but the naphthyl moiety is slightly shifted (Figure 2C). This difference might derive from the NAD^+^ bound and unbound state of Sirt2, which also induces a significant rearrangement of the cofactor binding loop, particularly involving Phe96.

**Figure 2:**
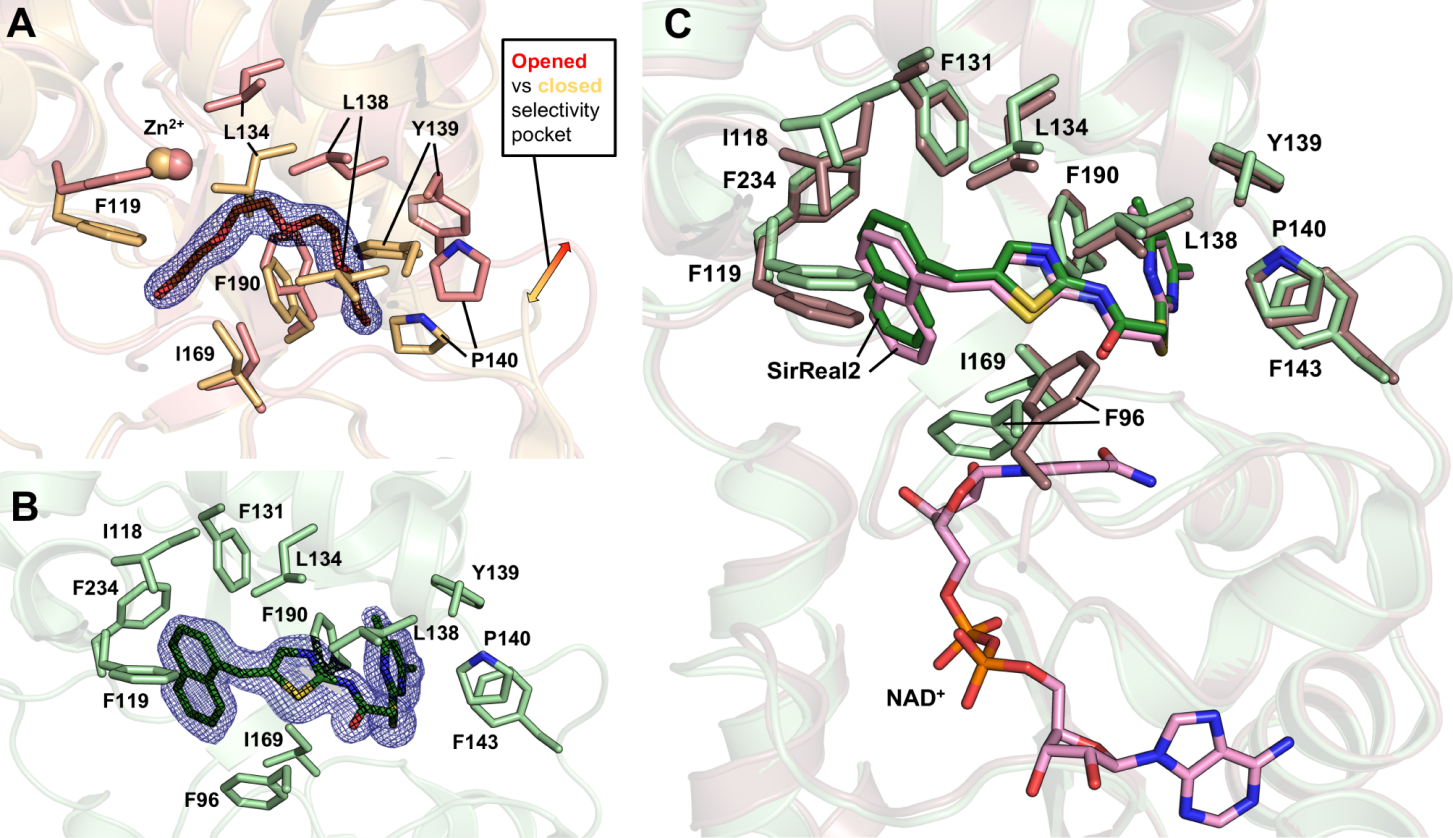
Crystal structures of Sirt2 apo (PDB 9FDR) and the Sirt2–**SirReal2** (PDB 9FDS) complex. (A) Superimposition of Sirt2 with an open selectivity pocket (Sirt2 = salmon, PEG = brown, PDB 9FDR), obtained via MMS experiments, and Sirt2 with closed selectivity pocket (light orange, PDB 3ZGO).^40^ The 2*F*_o_ – *F*_c_ map is depicted as a blue mesh and contoured at 1.0σ. The PEG molecule can only bind when the subpocket is present, otherwise it would clash with Leu134 and Leu138 from the hinge region. (B) Sirt2–**SirReal2** structure (Sirt2 = pale green, **SirReal2** = green, PDB 9FDS), obtained from soaking experiments. The 2*F*_o_ – *F*_c_ map is depicted as a blue mesh and contoured at 1.0σ. (C) Superimposition of Sirt2–**SirReal2** (Sirt2 = pale green, **SirReal2** = green), with Sirt2–**SirReal2**–NAD^+^ (Sirt2 = chocolate, **SirReal2** and NAD^+^ = light pink, PDB 4RMG).

### Fragment Library Screening by Fluorescence Polarization Assay and Structural Validation

The initial soaking experiments demonstrated that the Sirt2 apo crystals tolerate moderate DMSO levels of 10% (v/v) and that the crystal packing presents an accessible acyl-lysine channel. This confirmed the crystals’ suitability to generate structures with weak, fragment-like binders. Consequently, a screening of the Maybridge Ro3-fragment library, adhering to the ″rule of three″ (Molecular weight ≤ 300 Da; LogP ≤ 3.0; Number of H-bond acceptors/donors ≤ 3; Rotatable Bonds ≤ 3; polar surface area ≤ 60 Å^2^), was conducted using a fluorescence polarization assay (FPA). This biophysical binding assay is suitable for high-throughput testing of small inhibitor-fragments, as it does not rely on time-intensive enzymatic reactions. However, the FPA is mostly limited to inhibitors binding in the acyl-lysine channel, as the fluorescent SirReal-based TAMRA-probe (SirReal-TAMRA) needs to be displaced.^44–46^ Out of 1000 compounds, 22 hits could be identified that showed displacement of SirReal-TAMRA by at least 40% at 500 μM (see Table S1). Corroboration of the initial hits with differential scanning fluorimetry (DSF, also referred to as thermal shift assay) showed mostly weak thermal shifts of 0.5–1.0 °C at 1 mM (see Table S1), which is consistent with the binding of small fragments to proteins. Soaking experiments were performed with all hits, and three fragments – **1** (IC_50_ = 286 ± 10 μM, IC_50_ curve in Figure S4A), **2** (IC_50_ = 458 ± 54 μM, IC_50_ curve in Figure S4B) and **3** (IC_50_ = 565 ± 95 μM, IC_50_ curve in Figure S4C) – resulted in X-ray structures with sufficient electron density to enable model building. Consistent with their ability to compete with SirReal–TAMRA in the fluorescence polarization assay, all soaked fragments were identified to bind to sites (i.e., acyl-lysine channel and selectivity pocket) overlapping with the previously identified binding sites of triazole-based SirReals,^23,42^ such as SirReal–TAMRA. Although ligand binding to an allosteric site, such as the Zn^2+^-binding site, cannot be completely ruled out for the remaining 19 fragment hits for which no X-ray costructure was obtained, such an allosteric binding is rather unlikely since no Sirt2 inhibitor targeting the Zn^2+^-binding site is known to date.

The Sirt2–**1** fragment-inhibitor complex (Figure 3A, PDB 9FDU, final resolution of 1.55 Å) exhibits a so far unobserved rearrangement of amino acids 92–97 of the flexible cofactor binding loop, narrowing the acyl-lysine channel. Phe96 orients in a unique way that allows an orthogonal π–π-stacking network with Phe190, which forms π–π-stacking-interactions with the pyridine part of compound **1**. Furthermore, this reorientation results in H-bond interactions of −NH from the thioamide of **1** to the main chain C=O of Pro94 and Leu138. The −C=S residue of the thioamide forms a weak hydrogen bond network with a water molecule and the main chain −NH of Phe96 and C=O of Pro94. As a result, the acyl-lysine channel is blocked by Phe96 and the thioamide residue points toward the C-pocket of Sirt2, which usually is reserved for binding of the nicotinamide residue of NAD^+^ (Figure 3A, right panel). Interestingly, −CF_3_ further extends the selectivity pocket by a small subpocket reaching toward the Zn^2+^-binding domain.

**Figure 3:**
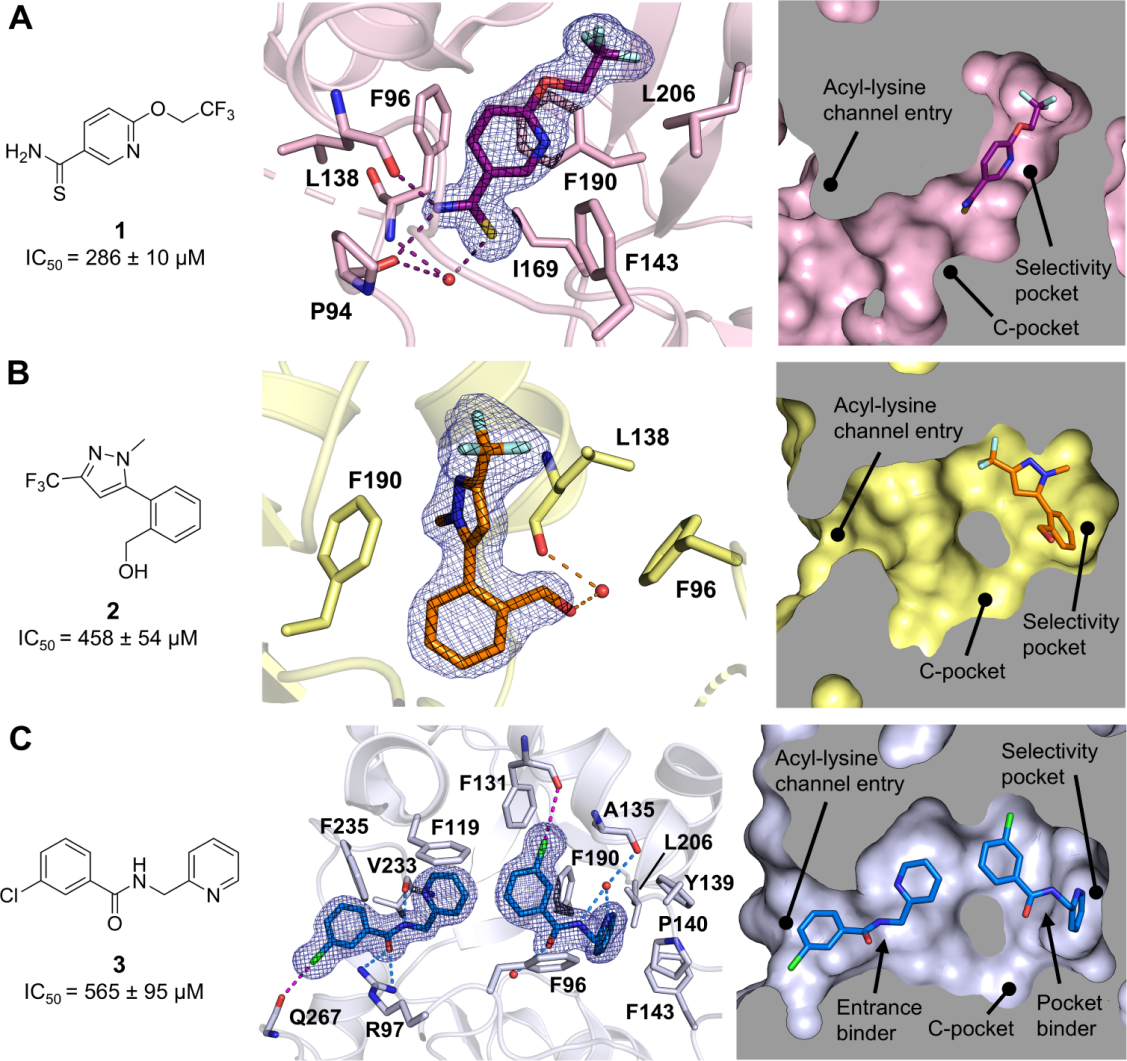
Chemical structures of the fragment-inhibitors and crystal structures of the three Sirt2-fragment complexes. (A) Sirt2–**1** complex (PDB 9FDU). *Left*: chemical structure of **1** with *in vitro* Sirt2 affinity. *Middle*: binding mode of **1** to Sirt2 (Sirt2 = light pink; **1** = dark violet). The hydrogen bonds of the thioamide −NH of **1** to Leu138 and Pro94 are shown as dashed dark violet lines. The 2*F*_o_ – *F*_c_ map is depicted as a blue mesh and contoured at 1.0σ. *Right*: cross-section surface representation of the binding mode of **1** inside the acyl-lysine channel of Sirt2. (B) Sirt2–**2** complex (PDB 9FDT). *Left*: chemical structure of **2** with *in vitro* Sirt2 affinity. *Middle*: binding mode of **2** to Sirt2 (Sirt2 = yellow; **2** = orange). The hydrogen bonds are shown as dashed orange lines and the water molecule is shown as a red sphere. The 2*F*_o_ – *F*_c_ map is depicted as a blue mesh and contoured at 1.0σ. *Right*: cross-section surface representation of the binding mode of **2** inside the acyl-lysine channel of Sirt2. (C) Sirt2–**3** complex (PDB 9FDW). *Left*: chemical structure of **3** with *in vitro* Sirt2 affinity. *Middle*: binding mode of the two molecules of **3** to Sirt2 (Sirt2 = blue white; **3** = blue). Both molecules of **3** exhibit well refined electron density, which indicates a simultaneous binding in the acyl-lysine channel. The hydrogen bonds are depicted as dashed blue lines, the halogen bonds are depicted as dashed magenta lines and the water molecules are shown as red spheres. The 2*F*_o_ – *F*_c_ map is depicted as a blue mesh and contoured at 1.0*σ. Right*: cross-section surface representation of the binding mode of **3** inside the acyl-lysine channel of Sirt2.

In the Sirt2–**2** fragment-inhibitor complex (Figure 3B, PDB 9FDT, final resolution of 1.60 Å), the phenylethoxy moiety of compound **2** is buried in the selectivity pocket of Sirt2 comprised of amino acids 138–143. The hydroxymethyl group forms a hydrogen bond with the main chain C=O of Leu138, mediated via a water molecule, potentially stabilizing this conformation in the crystal structure. The pyrazole moiety forms π–π-stacking interactions with Phe190.

The structure determination of the Sirt2–**3** fragment-inhibitor complex (Figure 3C, PDB 9FDW, final resolution of 1.60 Å) resulted in two molecules of compound **3** binding simultaneously inside the acyl-lysine channel of Sirt2, each with an occupancy of 1.0. This phenomenon has been previously described for the Sirt2–**EX243**–ADPR (PDB 5D7P) complex as well.^39^ Compound **3** exhibits similarities to a published benzamide Sirt2 inhibitor class,^47^ but it features an additional CH_2_ extension between the pyridine and the amide group. The pyridine moiety of the selectivity pocket binding molecule of compound **3** (referred to as “pocket binder”) is tightly locked between Tyr139 and Phe190 via π–π-stacking interactions. Additionally, it is further embedded in the pocket by Pro140, Phe143 and Leu206. A water molecule plays a pivotal role in the formation of a hydrogen bonding network, serving as a bridge between the main chain carbonyl of Ala135, the amide −NH, and pyridine nitrogen of the “pocket binder”. A second water molecule forms a hydrogen bond interaction with the carbonyl group of the amide moiety of the “pocket binder”. The chlorophenyl residue forms weak π–π-stacking interactions with Phe96 and a halogen bond to the main chain C=O of Phe131. The molecule of compound **3** binding inside the acyl-lysine channel entry (referred to as “entrance binder”), is distinguished by its reliance on hydrogen bond interactions rather than on π–π-stacking. The amide of compound **3** forms a hydrogen bond with the side chain of Arg97 and the main chain C=O of Val233. The interaction of an amide −NH with the main chain C=O of Val233 is a common feature of Sirt2 inhibitors,^33^ as well as for natural substrates of Sirt2.^9,22^ The “entrance binder” further forms a halogen bond with the main chain C=O of Gln267.

To explore a more physiological state of inhibitor binding that potentially involves NAD^+^ interaction, Sirt2 was soaked with compounds **1**, **2**, or **3** and NAD^+^, simultaneously. The resulting Sirt2 structures in complex with compounds **1** or **3** and NAD^+^ displayed electron density at the expected binding sites, but the occupancy and overall structure quality were insufficient for accurate model building. However, a structure was successfully solved for the Sirt2–**2**–NAD^+^ complex (Figure 4A, PDB 9FRU, final resolution of 2.00 Å). Initially, compound **2** and NAD^+^ (10 mM, pH 7.0) were soaked for 24 h. The well-defined electron density at both the A- and B-pockets revealed an occupiable NAD^+^ binding site in the Sirt2 apo crystals. However, the absence of electron density for NAD^+^’s nicotinamide residue at the C-pocket indicated a near-complete hydrolysis to ADPR (data not shown). Consequently, the NAD^+^ concentration was increased to 100 mM and the soaking duration reduced to 1 h. Despite the crystals’ limited tolerance to high NAD^+^ concentrations, leading to rapid dissolution and slight resolution loss, the structure still demonstrated NAD^+^ binding (Figure 4A) albeit with reduced electron density at the nicotinamide moiety and missing density for most of the cofactor-binding loop (amino acids 98–108). Compound **2** remains well-refined and aligned with the Sirt2–**2** structure (PDB 9FDT), indicating that NAD^+^ binding does not alter the inhibitor’s orientation (Figure 4B). Additionally, Phe96 flips inside the acyl-lysine channel to allow π–π stacking with both the nicotinamide of NAD^+^ and the pyrazole moiety of compound **2**. These findings confirm the accessibility of the cofactor-binding pocket for NAD^+^ (and ADPR), highlighting the potential of our approach for future soaking studies with NAD^+^-pocket binders.

**Figure 4:**
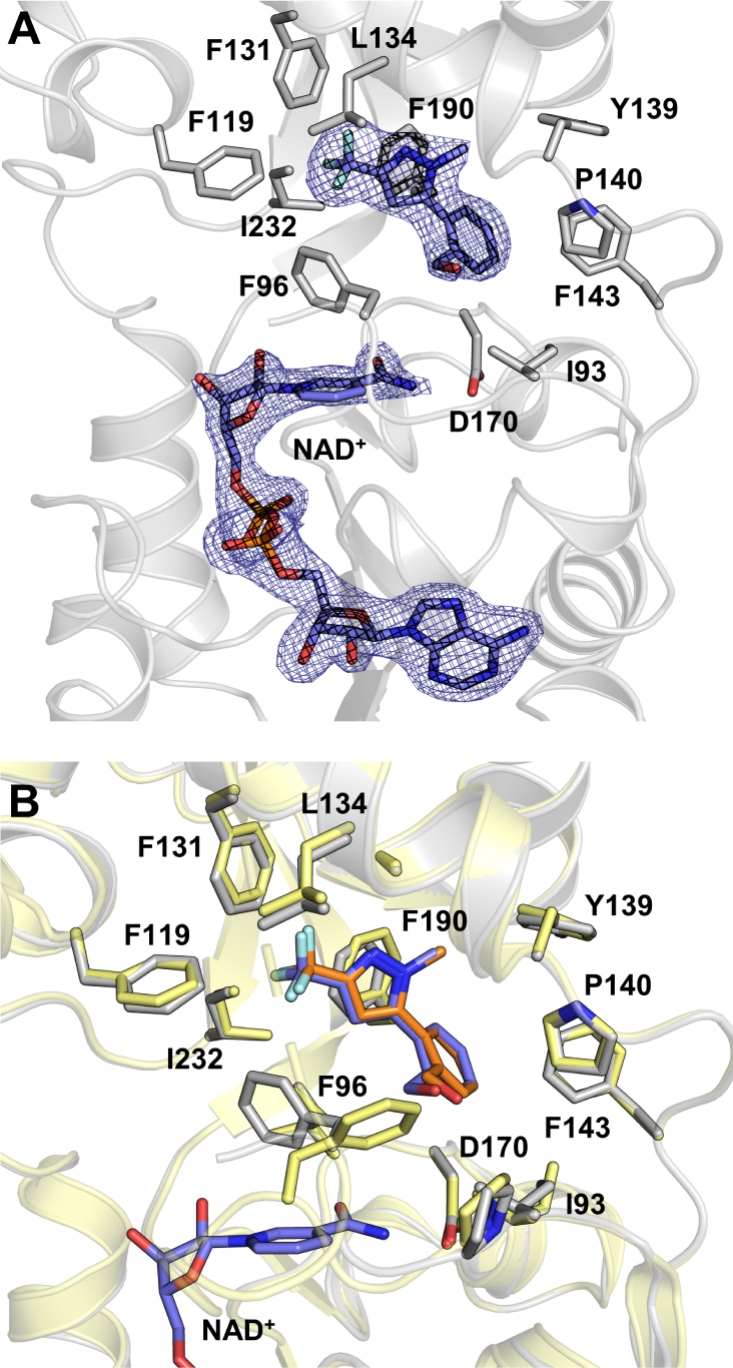
Crystal structure of Sirt2 in complex with **2** and NAD^+^(PDB 9FRU). (A) Sirt2–**2**–NAD^+^ complex (Sirt2 = gray; **2** and NAD^+^ = slate). The 2*F*_o__o_ – *F*_c_ map is depicted as a blue mesh and contoured at 1.0σ. (B) Superimposition of the Sirt2–**2**–NAD^+^ and the Sirt2–**2** complex (Sirt2 = yellow; **2** = orange). NAD^+^ binding does not affect the binding orientation of **2** but rearranges Phe96, which can form π–π-stacking with the nicotinamide moiety of NAD^+^ and the methyl-pyrazole of **2**.

### Binding to the Acyl-Lysine Channel Entry by the Peptide-Based Inhibitor KT9

To also demonstrate the suitability of the crystals for soaking molecules that target the acyl-lysine channel entry, we generated a Sirt2 structure in complex with the peptide-based inhibitor **KT9** (IC_50_ = 1.27 μM), containing a 3-((3,4-dichlorophenyl)­thio)­butanamide as acyl-lysine channel binding moiety (Figure 5, PDB 9FDX).^48^ **KT9** was soaked as an epimeric mixture, but the electron density map revealed the exclusive presence of the molecule containing the (*R*)-3-((3,4-dichlorophenyl)­thio)­butanamide moiety in the crystal structure. The 3,4-dichlorophenyl group is not oriented toward the selectivity pocket, but forms strong π–π interactions with Phe119 instead and, to a lesser extent, with Phe96. Additionally, Arg97 forms hydrogen bonds with the amide C=O of **KT9**. Notably, in the presence of NAD^+^, reported kinetic data indicate partial noncompetitive inhibition by **KT9**.^48^ However, the crystal structure shows that **KT9** adopts an orientation incompatible with simultaneous NAD^+^ binding due to predicted close contacts with the ribose moiety of NAD^+^ (Figure S3B). These close contacts arise from the 3,4-dichlorophenyl group’s orientation toward Phe119, Phe190, and Phe234 of the Zn^2+^-binding domain, which pushes the lysine residue of **KT9** toward the NAD^+^ binding pocket. Possibly, the crystal structure represents only one of multiple conformations in which **KT9** can bind to Sirt2. According to previous docking results, the 3,4-dichlorophenyl group can flip over inside the acyl-lysine channel, then pointing toward the selectivity pocket and allow NAD^+^ binding.^48^

**Figure 5:**
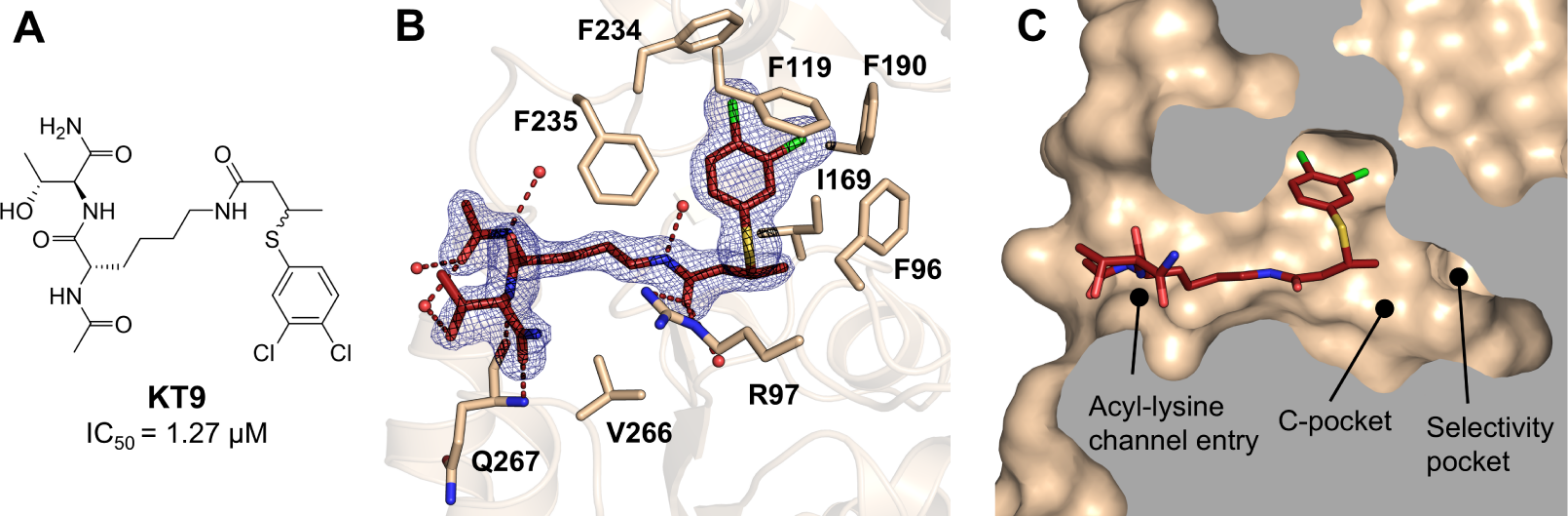
Crystal structure of the Sirt2–**KT9** complex (PDB 9FDX). (A) Chemical structure of **KT9** with *in vitro* affinity toward Sirt2.^48^ (B) Binding mode of **KT9** to Sirt2 (Sirt2 = wheat, **KT9** = firebrick). The hydrogen bonds are depicted as dashed firebrick lines and the water molecules are shown as red spheres. The 2*F*_o_ – *F*_c_ map is depicted as a blue mesh and contoured at 1.0σ. (C) Cross-section surface representation of Sirt2–**KT9** highlights the binding mode of **KT9** at the acyl-lysine channel entrance and its 3,4-dichlorophenyl residue pointing toward the Zn^2+^-binding domain.

## Discussion and Conclusion

As the growing number of newly identified Sirt2 protein substrates increasingly implicates a crucial role of Sirt2 in a wide range of diseases, the development of structurally diverse and selective Sirt2 inhibitors is important to understand these processes at the molecular level and for generating promising drug candidates. In this study, we developed a method to rapidly obtain high-quality Sirt2-inhibitor complex structures using Sirt2 apo crystals, which are well suited for high-throughput soaking experiments. The resulting crystal structures enabled the validation of initial fragment hits and, more importantly provided novel insights into the binding modes of these small molecules, which will aid in the structure-based optimization of new Sirt2 inhibitor scaffolds. The Sirt2 apo crystals allow targeting of the entire acyl-lysine channel, as well as the selectivity pocket and NAD^+^ binding site, which makes them widely applicable. It offers a distinct advantage over the current Sirt2 apo structure (PDB 3ZGO, see Figure S2A) and the Sirt2–ADPR system (PDB 5D7O, see Figure S2B), which both exhibit unfavorable crystal contacts for soaking experiments. Our crystallization approach exemplified for Sirt2 can be applied to other drug targets with pockets accessible only in specific induced conformations. Crystallizing apo proteins in an “induced fit” state through seeding with a ligand occupied protein in that state but with the ligand being absent in the final structure may enhance and accelerate structure-based drug discovery. The unique orientation of compound **1** suggests that an extension of the inhibitor toward the C-pocket of the NAD^+^ binding site could potentially result in improved affinities and a novel approach for Sirt2 inhibition, as the NAD^+^ binding site has not yet been addressed by inhibitors. For compound **3**, it is challenging to determine a priori whether one of the two molecules is a crystallization artifact or both binding positions are occupied in the inhibited Sirt2. The high occupancy and the fact that there are no interactions with neighboring Sirt2 molecules in the crystal structure seem to support the latter hypothesis. It is worthwhile to consider potential linkages between the two molecules, even if the aromatic residues are not optimally aligned for this procedure. Further synthesis efforts could potentially clarify these questions.

In summary, our highly versatile approach for obtaining high-quality Sirt2-inhibitor complex structures using Sirt2 apo crystals can further leverage the structure-based development of novel Sirt2 inhibitors, which will contribute to a deeper understanding of Sirt2 biology and its suitability as a drug target and has implications for other targets with ligand induced structural changes.

## Experimental Section

### Materials and Methods

Reagents and solvents were purchased from commercial suppliers (Carl Roth, ITW Reagents, Serva, Sigma-Aldrich) and used without further purification. The Maybridge Ro3 Fragment library and compound hits were purchased from Thermo Fisher Scientific. NAD^+^ was purchased from Carl Roth and **SirReal2** was purchased from MedChemExpress. **Mz242** ^23^ and **KT9** ^48^ were synthesized as previously reported.

### Protein Expression and Purification

For assay experiments, human strep-tagged Sirt2_56–356_ was expressed and purified as previously described.^26,49^ For crystallization experiments, human Sirt2_56–356_ was expressed and purified as previously described with minor modifications summarized hereafter.^22^ His-tagged Sirt2_56–356_ was overexpressed in 2 × YT medium (5 g/L NaCl, 16 g/L tryptone, 10 g/L yeast extract) using strain E. coli BL21 Star (DE3) at 20 °C overnight. Overexpression was induced by addition of IPTG (isopropyl β-d-1-thiogalactopyranoside, final concentration of 1.0 mM) at an OD_600_ of 0.6–0.8. The His_10_-Tag was cleaved via TEV protease digestion in lysis buffer (50 mM Tris/HCl, 500 mM NaCl, 10% (v/v) glycerol, 0.5 mM TCEP, pH 8.0) at 4 °C for 36 h. The protein was then applied to a HisTrap HP column (5 mL, GE Healthcare) to obtain pure fractions of His_10_-Tag cleaved Sirt2_56–356_ in the flowthrough. For the last purification step a Superdex S75 26/600 gel filtration column equilibrated with SEC buffer (20 mM HEPES, 150 mM NaCl, pH 7.5) was used.

### Crystallization and Soaking

Crystallization assays were prepared with the Oryx Nano pipetting robot (Douglas Instruments, East Garston, UK) using the vapor diffusion sitting drop method (MRC 2 Well UVP Plate, SWISSCI, Buckinghamshire, UK) at 20 °C.

Crystals of the Sirt2–**Mz242** complex were obtained as previously described.^42^ Briefly, Sirt2_56–356_ (13.2 mg/mL) was incubated with 1.8 mM **Mz242** (100 mM stock in DMSO, 1.8% (v/v) final DMSO concentration) on ice for 1 h prior to crystallization. The solution was centrifuged at 4 °C for 10 min to remove precipitates. Crystals of the Sirt2–**Mz242** complex formed after 20–30 days in wells containing 0.30 μL of protein solution and 0.30 μL of reservoir solution with 25% (w/v) PEG 3,350 in 0.1 M Bis-Tris at pH 6.5.

For Sirt2–**SirReal2**–NAD^+^ crystallization, Sirt2_56–356_ (11.4 mg/mL) was incubated with 3.3 mM SirReal2 (100 mM stock in DMSO, 3.3% (v/v) final DMSO concentration) and 10 mM β-NAD^+^ (in SEC buffer) on ice for 1 h prior to crystallization. The solution was centrifuged at 4 °C for 10 min to remove precipitates. Crystals formed after 3 days in wells containing 0.20 μL of protein solution and 0.40 μL of reservoir solution containing 27% (w/v) PEG 2,000 MME in 0.1 M Bis-Tris at pH 6.5.

Apo Sirt2_56–356_ crystals (11–13 mg/mL) formed after 1 day and reached maximum size by 5 days in wells with 0.30 μL of protein solution, 0.10 μL of Sirt2–**Mz242** or Sirt2–**SirReal2**–NAD^+^ seeding solution, and 0.25 μL of reservoir solution containing 22–32% (w/v) PEG 3,350 in 0.1 M HEPES, pH 7.25–8.0. The seeding solution was prepared by mixing crystals from the well with 500 μL of a solution identical to the reservoir, followed by vortex mixing with a Teflon seed bead to generate microcrystals. Once formed, apo Sirt2 crystals can be used as seeds for subsequent crystallization trials.

DMSO tolerance of apo Sirt2 crystals was tested by soaking crystals in reservoir solution mixed with 10% (v/v) DMSO for 24 h. For inhibitor soaking, a solution containing 32% (w/v) PEG 3,350, 0.1 M HEPES, pH 7.25–8.0 was mixed with 10% (v/v) inhibitor solution (in DMSO) to a final concentration of 5–100 mM, based on inhibitor affinity and solubility (**SirReal2** = 10 mM, **1** = 100 mM, **2** = 100 mM, **3** = 100 mM, **KT9** = 5 mM). Apo Sirt2 crystals were soaked in this mixture for 24 h, cryoprotected with reservoir solution containing 10% (v/v) DMSO, mounted on a nylon loop, and flash-cooled in liquid nitrogen. When NAD^+^ was included in the soaking solution (SEC buffer, pH 7.5, 100 mM final concentration), the soaking duration was limited to 1 h to prevent crystal dissolution.

### Data Collection and Structure Determination

X-ray diffraction data for the Sirt2 apo (PDB 9FDR), Sirt2–**SirReal2** (PDB 9FDS), Sirt2–**1** (PDB 9FDU), Sirt2–**2** (PDB 9FDT), Sirt2–**3** (PDB 9FDW), and Sirt2–**KT9** (PDB 9FDX) complexes were collected on the ID30B beamline at the European Synchrotron Radiation Facility (ESRF, Grenoble, France) using an EIGER2 X 9M detector. Data for the Sirt2–**2**–NAD^+^ (PDB 9FDR) complex was collected on the BM07 beamline at the ESRF with a PILATUS 6M detector. The data sets were processed with autoPROC^50^ and scaled using Aimless.^51^ The structures were solved by molecular replacement with Phaser^52^ using the Sirt2–**Mz242** complex (PDB 8OWZ)^42^ as the search model. Models were built and refined iteratively in COOT^53^ and either REFMAC^54^ or Phenix.refine.^55^ Restraints for the ligands were generated with the grade Web Server (Global Phasing Ltd., UK). Electron density was well-defined for all ligands. Final structures were validated using MolProbity.^56^ All data collection and refinement statistics are reported in Tables S2 and S3.

### Fluorescence Polarization Assay

Fluorescence polarization (FP) assays were conducted in a black 384-well microplate (OptiPlate-384 F, PerkinElmer) with a total volume of 20 μL and a final DMSO concentration of 5% (v/v), following a published protocol.^33,44^

A mixture of 14 μL Sirt2_56–356_ (200 nM final concentration) in assay buffer (50 mM Tris-HCl, 137 mM NaCl, 2.7 mM KCl, 1 mM MgCl_2_, 1 mg/mL BSA, 0.05% CHAPS, pH 8.0) and 1 μL of potential inhibitor (final concentrations: 2 mM, 500 μM, 125 μM) was incubated at 37 °C and 350 rpm for 10 min. For IC_50_ determination, dilution series of the ligand (20× concentrated in DMSO) were prepared. Blank controls contained 19 μL of assay buffer and 1 μL DMSO. Negative controls (*P*_neg_) used 1 μL **Mz242** (20 μM final concentration), while positive controls (P_pos_) replaced the inhibitor with 1 μL DMSO. After incubation, 5 μL of SirReal-TAMRA (40 nM final concentration, prepared from a 10 mM DMSO stock) was added, followed by further incubation at 37 °C and 350 rpm for 30 min. Fluorescence intensities were measured with an EnVision plate reader (PerkinElmer, optical module – BODIPY TMR FP, excitation filter – FP 531 nm, emission filter 1 – FP p-pol 595 nm, emission filter 2 – FP s-pol 595 nm). Inhibition was calculated using the equation reported below (*P*_I_, *P*_neg_, and *P*_pos_ are FP values of samples, positive control, and negative control, respectively).

$$I=100\times \left(1-\left(\frac{P_{I}-P_{neg}}{P_{pos}-P_{neg}}\right)\right)\%$$

### Fluorescence Thermal Shift Assay

Fluorescence thermal shift assays were performed in white 96-well plates (Hard-Shell PCR Plates, BioRad, USA) with a total volume of 20 μL and a final DMSO concentration of 5% (v/v), following a published protocol.^22,33^

A mixture of 10 μL Sirt2_56–356_ (6.0 μM final concentration) and SYPRO Orange (1:1.25 final dilution from a 1:5000 stock, Sigma-Aldrich, Germany) in assay buffer (25 mM Tris-HCl, 150 mM NaCl, 1 mM DTT, pH 8.0) was combined with 10 μL of potential inhibitor (final concentrations of 2.0 or 1.0 mM) and incubated at 25 °C and 350 rpm for 5 min. Fluorescence intensity was recorded during a temperature gradient of 1 °C per 20 s from 25 to 95 °C using a real-time PCR machine (C1000 Touch Thermal Cycler, CFX96 Real-Time System, BioRad, USA).

Melting temperatures were determined using GraphPad Prism, following a published procedure.^57^

## Supplementary Material



















## Data Availability

Additional tables and figures can be found in the Supporting Information. Atomic coordinates and structure factors for Sirt2 apo (PDB 9FDR), Sirt2–**SirReal2** (PDB 9FDS), Sirt2–**1** (PDB 9FDU), Sirt2–**2** (PDB 9FDT), Sirt2–**3** (PDB 9FDW), Sirt2–**KT9** (PDB 9FDX) and Sirt2–**2**–NAD^+^ (PDB 9FRU) have been deposited in the Protein Data Bank (www.rcsb.org). The authors will release the atomic coordinates upon article publication.

## Notes

Hits from the Ro3-Maybridge fragment library screening performed on Sirt2 (Table S1); crystallographic data collection and refinement statistics of the Sirt2–Inhibitor complexes (Tables S2 and S3); chemical structures of **EX527**, the 1,**2,4-oxadiazole** inhibitor and the thioamide-based peptide (Figure S1); structure and superimposition of Sirt2 apo and Sirt2–ADPR (Figure S2); superimposition of Sirt2 apo with Sirt2–**Mz242** and Sirt2–**KT9** with NAD^+^ (Figure S3); IC_50_ curves of compounds **1**–**3** in a previously reported Sirt2 binding assay based on fluorescence polarization (Figure S4) (PDF)
Molecular formula strings for the tested compounds (CSV)
Crystal structure of Sirt2 apo (CIF)
Crystal structure of Sirt2 in complex with **SirReal2** (CIF)
Crystal structure of Sirt2 in complex with **1** (CIF)
Crystal structure of Sirt2 in complex with **2** (CIF)
Crystal structure of Sirt2 in complex with **3** (CIF)
Crystal structure of Sirt2 in complex with **2** and NAD^+^ (CIF)
Crystal structure of Sirt2 in complex with **KT9** (CIF)

## Abbreviations

ADPR: Adenosine diphosphate ribose
ARF6: ADP-ribosylation factor 6
Cbz: benzyloxycarbonyl
DSF: differential scanning fluorimetry
FOXO3: Forkhead box O3
FP: fluorescence polarization
FPA: fluorescence polarization assay
FTSA: Fluorescence thermal shift assay
GAPDH: Glyceraldehyde 3-phosphate dehydrogenase
HEPES: 4-(2-hydroxyethyl)-1-piperazineethanesulfonicacid
IC_50_: half maximal inhibitory concentration
IPTG: isopropyl β-D-1-thiogalactopyranoside
KRas: Kirsten rat sarcoma virus protein
MME: monomethyl ether
MMS: microseed matrix screening
NAD^+^: Nicotinamide adenine dinucleotide
OD_600_: optical density at 600 nm
PEG: polyethylene glycol
PROTAC: proteolysis targeting chimera
RalB: Ras-related protein Ral-B
rmsd: root mean-square deviation
Ro3: Rule of three
SAR: structure–activity relationship
SirReal: Sirtuin rearranging ligand
Sirt: Sirtuin
TAMRA: Carboxytetramethylrhodamine
TCEP: Tris­(2-carboxyethyl)­phosphine
TEV: tobacco etch virus
TM: thiomyristoyl
Tris: Tris­(hydroxymethyl)­aminomethane
