# Supplementary material for: Efficient Crystallization of Apo Sirt2 for Small-Molecule Soaking and Structural Analysis of Ligand Interactions
Source: J Med Chem. 2025 May 20;68(11):10771–80. doi: 10.1021/acs.jmedchem.4c02896 (PMC12169609; doi:10.1021/acs.jmedchem.4c02896)
Supplement: Supplementary file 1 [file jm4c02896_si_001.pdf]

Supporting Information

Efficient Crystallization of Apo Sirt2 for Small Molecule Soaking and Structural Analysis of Ligand Interactions

Florian Friedrich,[a] *Matthias Schiedel,*[b] *Sören Swyter,*[a] *Lin Zhang,*[c,d] *Wolfgang Sippl,*[e] *Mike Schutkowski,*[f] *Oliver Einsle,*[c] *Manfred Jung* [a,*]

[a] Institute of Pharmaceutical Sciences, University of Freiburg, 79104 Freiburg, Germany

[b] Institute of Medicinal and Pharmaceutical Chemistry, Technische Universität Braunschweig, 38106 Braunschweig, Germany

[c] Institute of Biochemistry, University of Freiburg, 79104 Freiburg, Germany

[d] Faculty of Synthetic Biology, Shenzhen University of Advanced Technology, Shenzhen, 518107, China

[e] Department of Medicinal Chemistry, Institute of Pharmacy, Martin-Luther-University of Halle-Wittenberg, 06120 Halle, Germany

[f] Department of Enzymology, Charles Tanford Protein Center, Institute of Biochemistry and Biotechnology, Martin-Luther-University Halle-Wittenberg, 06120 Halle, Germany

*Corresponding author: Prof. Dr. Manfred Jung, Institute of Pharmaceutical Sciences, University of Freiburg, 79104 Freiburg, Germany

E-mail: manfred.jung@pharmazie.uni-freiburg.de

TABLE OF CONTENTS

S3 Figure S1

S4 Figure S2

S5 Figure S3

S5 Figure S4

S6 Table S1

S8 Table S2

S9 Table S3

S10 Supplementary References

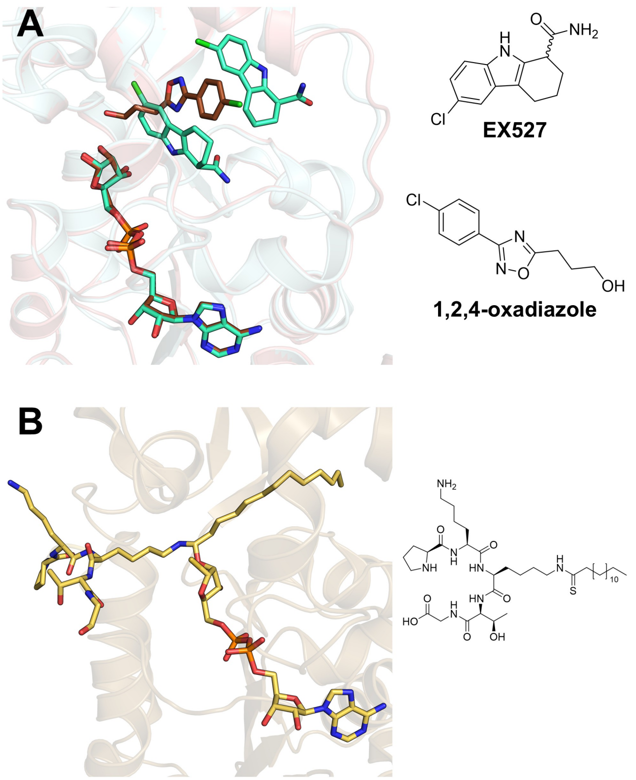

Figure S1: (**A**) *Left*: Superimposed structures of Sirt2-**EX527**-ADPR (Sirt2 = pale cyan, **EX527** and ADPR = green cyan, PDB 5D7P)[1] and Sirt2-**1,2,4-oxadiazole**-ADPR (Sirt2 = raspberry, 1,2,4-oxadiazole and ADPR = brown, PDB 5MAR)[2], obtained *via* soaking of Sirt2-ADPR crystals. *Right*: Chemical structures of **EX527** and the **1,2,4-oxadiazole** inhibitor. (**B**) *Left*: Sirt2 mechanism-intermediate obtained *via* soaking of NAD^+ in Sirt2-TM crystals (Sirt2 = gold, thioamide-based peptide = yellow orange, PDB 4X3O). *Right*: Chemical structure of the thioamide-based peptide.

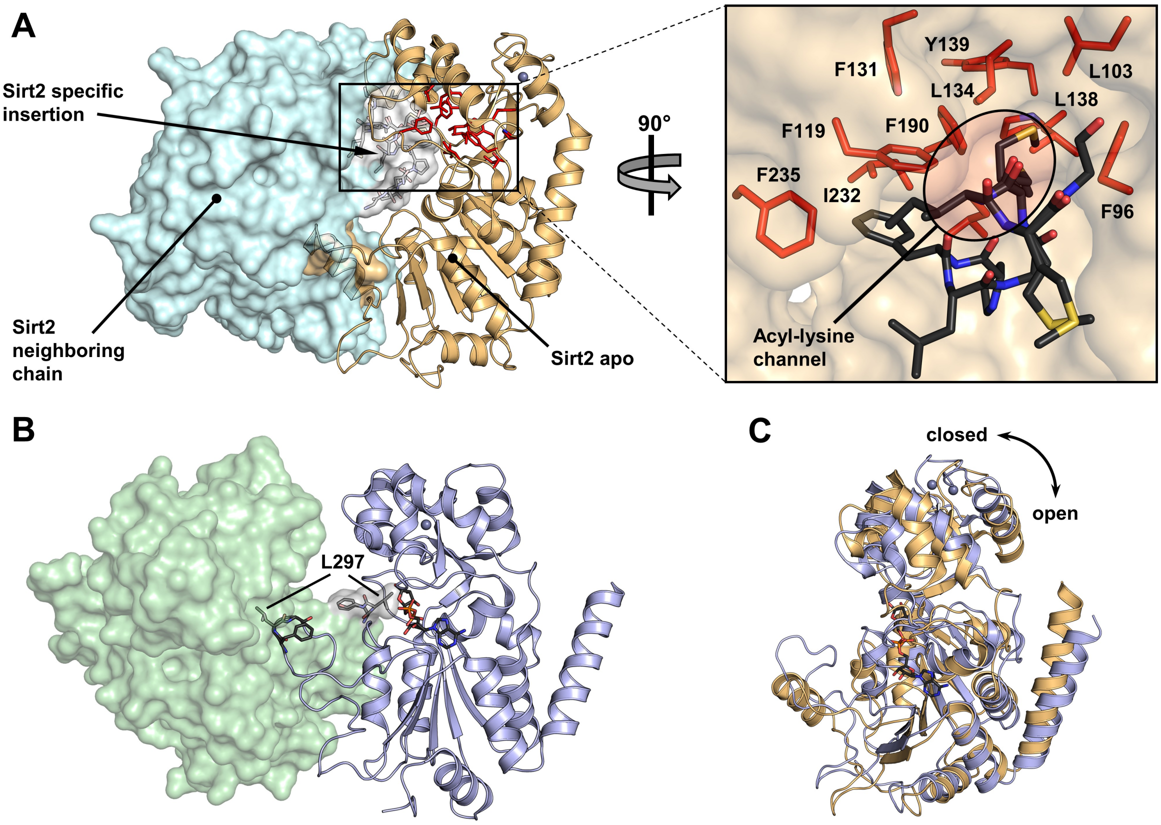

Figure S2: (**A**) Structure of Sirt2 apo (light orange cartoon representation, chain C, PDB 3ZGO)[3] displayed with its symmetry mate (pale cyan surface representation, chain A) in the crystal structure. The Sirt2-specific insertion composing of amino acids 291 – 305 (shown as black sticks and gray surface) forms essential crystal contacts between the two Sirt2 monomers, which leads to the blocking of the acyl-lysine channel (indicated as red sticks). (**B**) The structure of Sirt2-ADPR (chain A: light blue cartoon representation, chain B: pale green surface representation, ADPR: black sticks, PDB 5D7O)[1] reveals that Leu297 of Sirt2 "chain A" binds inside the acyl lysine channel entry of Sirt2 "chain B" and vice versa. This binding results in a Zn^{2+}-binding domain closure and partial blocking of the channel. (**C**) Superimposition of Sirt2 apo (light orange, opened state, PDB 3ZGO) and Sirt2-ADPR (light blue, closed state, 5D7O) shows the Zn^{2+}-binding domain flexibility.

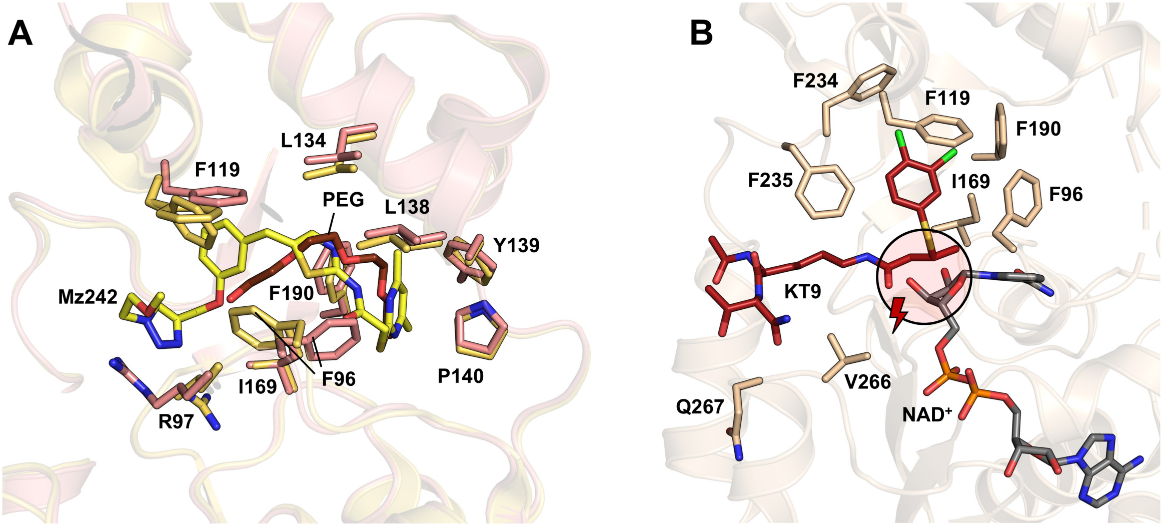

Figure S3: (**A**) Superimposition of Sirt2 apo (salmon, PDB 9FDR) with Sirt2-**Mz242** (Sirt2 = yellow-orange, **Mz242** = yellow, PDB 8OWZ).[4] All amino acids that are involved in the formation of the selectivity pocket align very well. However, Phe96 and Arg97 from the cofactor binding loop show a different orientation, resulting from the presence or absence of **Mz242**. (**B**) Superimposition of Sirt2-**KT9** (**Sirt2** = wheat, **KT9** = firebrick, PDB 9FDX) with NAD^+ (gray) from PDB 4RMG. In the presented **KT9** orientation, the close distance of the amide C=O of **KT9** and the ribose moiety of NAD^+ (highlighted as black circle) would result in steric clashes that prevent simultaneous binding.

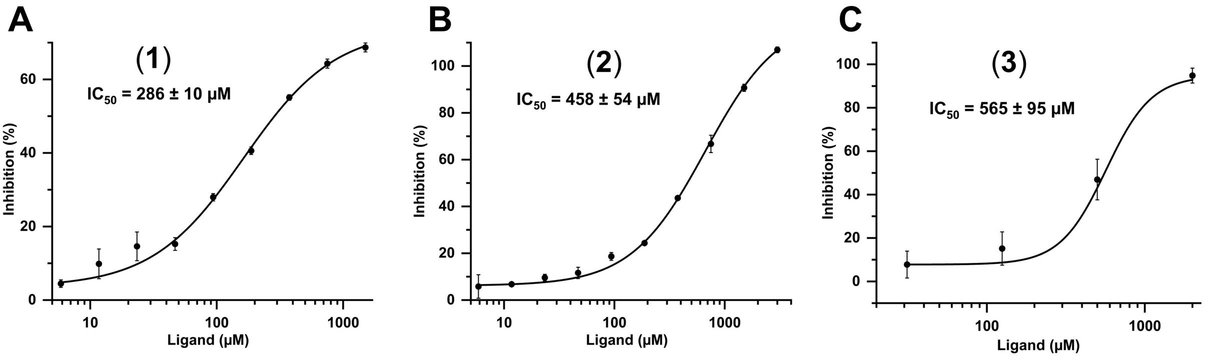

Figure S4: IC_{50} curves of compounds **1-3** in a previously reported Sirt2 binding assay based on fluorescence polarization.[5] At concentrations exceeding 1000 µM, **1** started to precipitate, thus limiting the maximum inhibition to 70%.

Table S1: Hits from the Ro3-Maybridge fragment library screening performed on Sirt2. Successfully soaked compounds are marked in green. n.d. = not determined.

No.	ID	Structure	FP-Assay<br>Inhibition (%) at:<br>2000 µM<br>500 µM<br>125 µM	FTS-Assay<br>Thermal shift at:<br>2000 µM<br>1000 µM
**1**	**(1)**	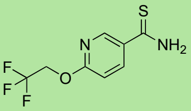	74.2 ± 3.1<br>55.4 ± 4.3<br>26.5 ± 5.8	0.6 °C<br>0.3 °C
**2**	**(2)**	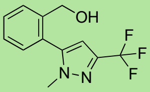	104.1 ± 2.9<br>47.4 ± 4.6<br>-1.6 ± 5.4	0.7 °C<br>0.7 °C
**3**	**(3)**	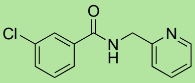	94.8 ± 3.4<br>46.9 ± 9.3<br>15.1 ± 7.7	- 0.2 °C<br>0.5 °C
**4**	**-**	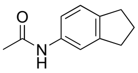	78.5 ± 7.5<br>61.1 ± 6.5<br>32.8 ± 4.3	0.1 °C<br>1.1 °C
**5**	**-**	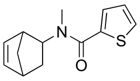	43.8 ± 10.1<br>10.3 ± 6.0<br>-7.4 ± 8.3	0.7 °C<br>0.5 °C
**6**	**-**	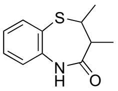	101.0 ± 4.7<br>59.9 ± 7.9<br>20.2 ± 2.9	n.d.<br>1.5 °C
**7**	**-**	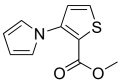	76.2 ± 1.8<br>46.9 ± 4.3<br>11.5 ± 5.5	0.5 °C<br>0.8 °C
**8**	**-**	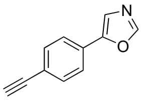	n.d.<br>61.2 ± 3.3<br>19.1 ± 4.6	0.0 °C<br>0.9 °C
**9**	**-**	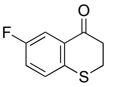	48.5 ± 5.7<br>39.5 ± 5.3<br>-10.6 ± 6.3	0.1 °C<br>1.0 °C
**10**	**-**	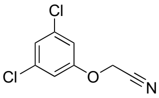	90.3 ± 14.7<br>53.9 ± 8.2<br>19.3 ± 10.5	- 0.0 °C<br>0.7 °C
**11**	**-**	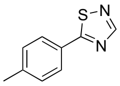	100.5 ± 4.5<br>52.5 ± 8.6<br>12.1 ± 5.5	0.5 °C<br>0.5 °C
**12**	**-**	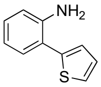	104.2 ± 3.2<br>66.4 ± 3.2<br>33.2 ± 10.3	0.0 °C<br>- 0.1 °C
**13**	**-**	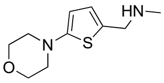	95.1 ± 3.5<br>49.3 ± 4.1<br>19.9 ± 3.1	n.d.<br>n.d.
**14**	**-**	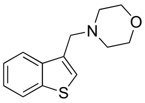	91.9 ± 13.9<br>42.5 ± 2.9<br>24.3 ± 3.5	0.7 °C<br>1.3 °C
**15**	**-**	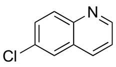	87.3 ± 6.8<br>36.2 ± 6.5<br>6.3 ± 7.4	0.2 °C<br>0.7 °C
**16**	**-**	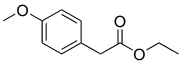	n.d.<br>50.5 ± 5.4<br>23.1 ± 3.9	0.4 °C<br>0.5 °C

**17**	**-**	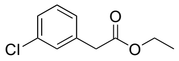	99.7 ± 4.6<br>63.8 ± 2.4<br>24.7 ± 4.0	0.9 °C<br>0.9 °C
**18**	**-**	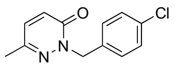	90.8 ± 2.8<br>41.9 ± 7.0<br>11.3 ± 3.2	0.1 °C<br>1.3 °C
**19**	**-**	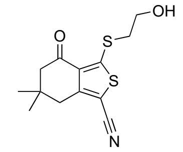	91.5 ± 1.7<br>40.9 ± 5.6<br>8.2 ± 4.9	- 0.7 °C<br>- 0.5 °C
**20**	**-**	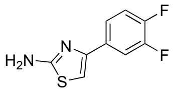	91.3 ± 3.2<br>38.8 ± 4.6<br>-0.9 ± 6.3	0.0 °C<br>- 0.4 °C
**21**	**-**	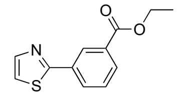	97.0 ± 6.2<br>91.4 ± 2.5<br>41.8 ± 5.9	- 0.1 °C<br>0.9 °C
**22**	**-**	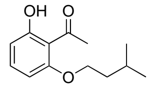	56.5 ± 2.8<br>74.2 ± 4.2<br>57.6 ± 7.3	1.6 °C<br>1.9 °C

Table S2: Crystallographic data collection and refinement statistics of the Sirt2-**Inhibitor** complexes.

Structure	Sirt2 apo	Sirt2-**SirReal2**	Sirt2-**1**	Sirt2-**2**
Beamline	ID30B	ID30B	ID30B	ID30B
**Data Collection**				
Space group	$P2_1$	$P2_1$	$P2_1$	$P2_1$
a, b, c (Å)	35.93, 73.25, 54.76	35.75, 73.56, 55.47	36.03, 72.91, 54.69	35.82, 73.18, 54.96
α, β, γ (deg)	90.00, 95.52, 90.00	90.00, 94.70, 90.00	90.00, 96.11, 90.00	90.00, 95.67, 90.00
Wavelength (Å)	0.8731	0.8731	0.8731	0.8731
Resolution (Å)	73.25 – 1.25 (1.27 – 1.25)	73.55 – 1.40 (1.42 – 1.40)	72.91 – 1.55 (1.58 – 1.55)	73.18 – 1.60 (1.63 – 1.60)
Total/unique no. of reflections	421,255 (77,852)	219,522 (49,786)	275,160 (40,442)	236,334 (34,589)
R_{merge}[a,b]	0.057 (1.082)	0.053 (1.003)	0.083 (1.344)	0.085 (1.379)
R_{pim}[a,c]	0.035 (0.674)	0.036 (0.696)	0.034 (0.566)	0.035 (0.553)
$CC_{1/2}$[a,d]	0.999 (0.614)	0.999 (0.602)	0.998 (0.687)	0.977 (0.669)
$I/\sigma(I)$[a]	12.2 (1.5)	12.9 (1.6)	10.6 (1.5)	10.1 (1.4)
Redundancy[a]	5.4 (5.5)	4.4 (4.7)	6.8 (6.5)	6.8 (7.2)
Completeness (%)[a]	99.9 (99.9)	88.4 (99.1)	99.2 (98.5)	92.8 (100)
**Refinement**				
No. of reflections used in refinement/test set	77,803 (7,741)	49,752 (4,748)	40,380 (3,977)	34,533 (3,690)
R_{work}[e]	0.179 (0.296)	0.174 (0.273)	0.189 (0.266)	0.189 (0.289)
R_{free}[f]	0.189 (0.325)	0.198 (0.273)	0.215 (0.285)	0.210 (0.342)
Number of Atoms[g]	2776	2684	2550	2487
protein	2412	2369	2302	2296
ligands	44	51	33	36
solvent	320	264	215	155
Average *B*-Factors ($Å^2$)	23.0	24.9	33.9	34.9
protein	21.7	24.0	33.2	34.6
ligands	36.5	29.3	41.7	41.3
solvent	30.5	32.2	39.8	37.7
RMS Deviations				
bonds (Å)	0.005	0.005	0.006	0.006
angles (deg)	0.85	0.82	0.85	0.87
Ramachandran plot (%)[h]				
favored	97.95	98.28	98.21	98.23
allowed	2.05	1.72	1.79	1.77
outliers	0	0	0	0
PDB accession code	**9FDR**	**9FDS**	**9FDU**	**9FDT**

[a] Values in parentheses refer to the highest-resolution shell of the data.

[b] $R_{merge} = \sum|I_h - \langle I_h\rangle|/\sum\langle I_h\rangle$; I_h = intensity measure for reflection h; $\langle I_h\rangle$ = average intensity for reflection h calculated from replicate data.

[c] $R_{pim} = \sum(1/(n-1))^{1/2}|I_h - \langle I_h\rangle|/\sum\langle I_h\rangle$; n = number of observations (redundancy).

[d] $CC_{1/2} = \sigma_\tau^2/(\sigma_\tau^2 + \sigma_\varepsilon^2)$, where σ_τ^2 is the true measurement error variance and σ_ε^2 is the independent measurement error variance.

[e] $R_{work} = \sum||F_o| - |F_c||/\sum|F_o|$ for reflections contained in the working set. $|F_o|$ and $|F_c|$ are the observed and calculated structure factor amplitudes, respectively.

[f] $R_{free} = \sum||F_o| - |F_c||/\sum|F_o|$ for reflections contained in the test set held aside during refinement.

[g] Per asymmetric unit.

[h] Assessed by MolProbity.

Table S3: Crystallographic data collection and refinement statistics of the Sirt2-**Inhibitor** complexes.

Structure	Sirt2-**3**	Sirt2-**KT9**	Sirt2-**2**-NAD$^+$
Beamline	ID30B	ID30B	BM07
**Data Collection**			
Space group	$P2_1$	$P2_1$	$P2_1$
a, b, c (Å)	35.83, 73.15, 54.95	36.12, 73.25, 55.18	35.87, 73.31, 54.98
α, β, γ (deg)	90.00, 95.70, 90.00	90.00, 95.17, 90.00	90.00, 95.53, 90.00
Wavelength (Å)	0.8731	0.8731	0.9795
Resolution (Å)	73.15 – 1.60 (1.63 – 1.60)	73.25 – 1.55 (1.58 – 1.55)	54.71 – 2.00 (2.05 – 2.00)
Total/unique no. of reflections	204,962 (34,166)	287,302 (41,529)	121,596 (17,792)
R_{merge}[a,b]	0.063 (1.104)	0.060 (0.702)	0.081 (0.954)
R_{pim}[a,c]	0.028 (0.478)	0.025 (0.290)	0.046 (0.390)
$CC_{1/2}$[a,d]	0.999 (0.664)	0.999 (0.865)	0.999 (0.746)
$I/\sigma(I)$[a]	14.3 (1.6)	15.3 (2.7)	16.3 (2.0)
Redundancy[a]	6.0 (6.1)	6.9 (6.8)	6.8 (6.9)
Completeness (%)[a]	91.7 (99.9)	99.9 (100)	92.7 (99.2)
**Refinement**			
No. of reflections used in refinement/test set	34,132 (3,701)	41,487 (4,127)	17,749 (1,589)
R_{work}[e]	0.187 (0.275)	0.178 (0.222)	0.195 (0.256)
R_{free}[f]	0.214 (0.322)	0.206 (0.263)	0.230 (0.314)
Number of Atoms[g]	2628	2624	2463
protein	2364	2336	2256
ligands	51	55	75
solvent	213	233	132
Average *B*-Factors (Å^2)	31.2	33.0	38.5
protein	30.6	32.4	38.2
ligands	33.0	39.5	45.7
solvent	36.9	38.0	40.5
RMS Deviations			
bonds (Å)	0.007	0.006	0.004
angles (deg)	0.92	0.85	0.70
Ramachandran plot (%)[h]			
favored	96.91	97.52	97.10
allowed	3.09	2.48	2.90
outliers	0	0	0
PDB accession code	**9FDW**	**9FDX**	**9FRU**

[a] Values in parentheses refer to the highest-resolution shell of the data.

[b] $R_{merge} = \sum|I_h - \langle I_h\rangle|/\sum\langle I_h\rangle$; I_h = intensity measure for reflection *h;* $\langle I_h\rangle$ = average intensity for reflection *h* calculated from replicate data.

[c] $R_{pim} = \sum(1/(n-1))^{1/2}|I_h - \langle I_h\rangle|/\sum\langle I_h\rangle$; n = number of observations (redundancy).

[d] $CC_{1/2} = \sigma_\tau^2/(\sigma_\tau^2 + \sigma_\varepsilon^2)$, where σ_τ^2 is the true measurement error variance and σ_ε^2 is the independent measurement error variance.

[e] $R_{work} = \sum||F_o| - |F_c||/\sum|F_o|$ for reflections contained in the working set. $|F_o|$ and $|F_c|$ are the observed and calculated structure factor amplitudes, respectively.

[f] $R_{free} = \sum||F_o| - |F_c||/\sum|F_o|$ for reflections contained in the test set held aside during refinement.

[g] Per asymmetric unit.

[h] Assessed by MolProbity.

Supplementary References

(1) Rumpf, T.; Gerhardt, S.; Einsle, O.; Jung, M. Seeding for Sirtuins: Microseed Matrix Seeding to Obtain Crystals of Human Sirt3 and Sirt2 Suitable for Soaking. *Acta Crystallogr. Sect. F Struct. Biol. Commun.* **2015**, *71* (12), 1498–1510. https://doi.org/10.1107/S2053230X15019986.

(2) Moniot, S.; Forgione, M.; Lucidi, A.; Hailu, G. S.; Nebbioso, A.; Carafa, V.; Baratta, F.; Altucci, L.; Giacché, N.; Passeri, D.; Pellicciari, R.; Mai, A.; Steegborn, C.; Rotili, D. Development of 1,2,4-Oxadiazoles as Potent and Selective Inhibitors of the Human Deacetylase Sirtuin 2: Structure–Activity Relationship, X-Ray Crystal Structure, and Anticancer Activity. *J. Med. Chem.* **2017**, *60* (6), 2344–2360. https://doi.org/10.1021/acs.jmedchem.6b01609.

(3) Moniot, S.; Schutkowski, M.; Steegborn, C. Crystal Structure Analysis of Human Sirt2 and Its ADP-Ribose Complex. *J. Struct. Biol.* **2013**, *182* (2), 136–143. https://doi.org/10.1016/j.jsb.2013.02.012.

(4) Sinatra, L.; Vogelmann, A.; Friedrich, F.; Tararina, M. A.; Neuwirt, E.; Colcerasa, A.; König, P.; Toy, L.; Yesiloglu, T. Z.; Hilscher, S.; Gaitzsch, L.; Papenkordt, N.; Zhai, S.; Zhang, L.; Romier, C.; Einsle, O.; Sippl, W.; Schutkowski, M.; Gross, O.; Bendas, G.; Christianson, D. W.; Hansen, F. K.; Jung, M.; Schiedel, M. Development of First-in-Class Dual Sirt2/HDAC6 Inhibitors as Molecular Tools for Dual Inhibition of Tubulin Deacetylation. *J. Med. Chem.* **2023**, *66* (21), 14787–14814. https://doi.org/10.1021/acs.jmedchem.3c01385.

(5) Swyter, S.; Schiedel, M.; Monaldi, D.; Szunyogh, S.; Lehotzky, A.; Rumpf, T.; Ovádi, J.; Sippl, W.; Jung, M. New Chemical Tools for Probing Activity and Inhibition of the NAD+-Dependent Lysine Deacylase Sirtuin 2. *Philos. Trans. R. Soc. B Biol. Sci.* **2018**, *373* (1748), 20170083. https://doi.org/10.1098/rstb.2017.0083.
